# Unraveling the interplay between phylogeny and chemical niches in epiphytic macrolichens

**DOI:** 10.1007/s00442-024-05641-9

**Published:** 2024-12-07

**Authors:** Yngvar Gauslaa, Jason Hollinger, Trevor Goward, Johan Asplund

**Affiliations:** 1 Faculty of Environmental Sciences and Natural Resource Management, Norwegian University of Life Sciences, P.O. Box 5003, 1432 Ås, Norway; 2 Herbarium, Department of Biology, Western Carolina University, Cullowhee, NC 28723 USA; 3 UBC Herbarium, Beaty Museum, University of British Columbia, Vancouver, BC V6T 1Z4 Canada; 4 Enlichened Consulting Ltd., 5369 Clearwater Valley Road, Upper Clearwater, BC V0E 1N1 Canada

**Keywords:** Canopy throughfall, Epiphytic lichens, Lecanorales, Lichen evolution, Peltigerales

## Abstract

This study aims to elucidate the connection between the phylogeny of epiphytic macrolichens and their chemical niches. We analyzed published floristic and environmental data from 90 canopies of *Picea glauca* x *engelmannii* across various forest settings in British Columbia. To explore the concordance between a principal coordinates analysis of the cladistic distance matrix and a global non-metric multidimensional scaling of the ecological distance matrix, we used Procrustean randomization tests. The findings uncover a robust association between large-scale macrolichen phylogeny and canopy throughfall chemistry. The high calcium-scores of the studied species effectively distinguished members of the Peltigerales from those of the Lecanorales, although parameters linked with Ca such as Mn, Mg, K, bark-, and soil-pH, may contribute to the niche partitioning along the oligotrophic—mesotrophic gradient. The substantial large-scale phylogenetic variation in the macrolichens’ Ca-scores is consistent with an ancient adaptation to specialized chemical environments. Conversely, the minor variation in Ca-scores within families and genera likely stems from more recent adaptation. This study highlights crucial functional and chemical differences between members of the Lecanorales and Peltigerales. The deep phylogenetic connection to the chemical environment underscores the value of lichens as transferable bioindicators for the chemical environment and emphasizes the importance of elucidating the intricate interplay between chemical factors and lichen evolution.

**Supplementary Information:**

The online version contains supplementary material available at 10.1007/s00442-024-05641-9.

## Introduction

The lichen consortium has a long history, dating back up to 480 million years (Lutzoni et al. 2018). The molecular clock data suggest a divergence time for the primarily lichenized class of Lecanoromycetes (subphylum Pezizomycotina) of 850–250 million years ago (Lücking et al. 2009). Nonetheless, only a few early lichen fossils are known, including a 415-million-year-old cyanolichen that resembles extant Peltigerales species, and a putative chlorolichen of the same age (Honegger et al. 2013; Lücking and Nelsen 2018). Puginier et al. (2022) recently suggested that green algae and early plants colonized terrestrial habitats more or less simultaneously, in both cases greatly benefitting from symbiotic associations with facilitating fungi and the development of genes involved in nutrient exchange. In this connection, it is interesting to note that major lichen diversification coincided with the emergence of vascular plants (Nelsen et al. 2020b). Lichen diversification within Peltigerales and Lecanorales (family Parmeliaceae) was rapid during the Cretaceous period (Amo de Paz et al. 2011; Widhelm et al. 2021) when angiosperm trees proliferated (Crane et al. 1995).

Unlike vascular plants, lichens have no roots. Instead, they absorb airborne elements primarily from diluted hydration sources such as rain, fog, and dew. This process is facilitated by efficient nutrient accumulation mechanisms (Nieboer et al. 1978). These adaptations, likely present early in the evolutionary history of lichens, were crucial for survival on e.g., barren land where nutrients were too low to support a dense vegetation. In addition to rain, fog, and dew, lichens may acquire nutrients to varying degree from their surroundings (Brown 1976; Nash 2008). By inhabiting the interface between plant surfaces and the atmosphere, the epiphytic lichens interact with a temporally changing canopy throughfall chemistry that varies between tree species (Lang et al. 1976; Knops et al. 1996; Van Stan and Pypker 2015), with markedly different elemental stoichiometry between angio- and gymnosperms (Sardans et al. 2016), the latter providing more acidic and Mn-rich stemflow and canopy throughfall (Asplund et al. 2015; Gauslaa et al. 2020). During the Cretaceous period, the arboreal angiosperms began to overtake the earlier dominance of gymnosperms, resulting in more balanced nutrient solutions for lichens on certain deciduous trees higher in base cations. The increasing variety of tree species led to more diverse canopy throughfall chemistry and likely resulted in significant evolutionary pressure, in effect forcing epiphytic lichens to adapt to one or the other chemical category of trees.

Recent studies have underscored the increasing awareness that forest structures significantly impact the phylogenetic diversity of epiphytic communities (Hurtado et al. 2019; Nascimento et al. 2021; Koch et al. 2022). However, the quantitative relationship between lichen phylogeny and their chemical niches remains largely unexplored, and numeric analyses of datasets containing both ecological and phylogenetic data are rare in literature (Legendre et al. 1994).

Here, we leverage chemical (Gauslaa et al. 2020) and floristic data (Gauslaa et al. 2021) from canopies of *Picea glauca* x *engelmannii* trees, located within or outside the dripzones of the angiosperm tree genus *Populus*. The study sites span a range of unmanaged forest types in southern British Columbia, which includes settings from base cation-rich to acidic Mn-rich canopy throughfall. This allows us to quantitatively investigate the relationship between chemical variables and the fungal phylogeny of the lichens reported in these earlier studies. Our approach is to construct a phylogenetic tree and a patristic distance matrix for the lichen mycobionts concerned, making it possible to identify the ecological parameters that best align with the phylogenetic clades. We hypothesize that chemical differences deeply rooted within the phylogeny suggest chemical adaptation early in ascomycetes evolution, while absence of deep rooting suggests a more rapid and simpler genotype change between the chemical specializations, consistent with a more recent origin.

## Materials and methods

### Study sites and canopy settings

Our research was carried out in the Clearwater Valley of south-central British Columbia (51° N, 120° W; 660–830 m a.s.l.). The soils are primarily derived from underlying peralkaline basalts laid down by volcanic eruptions that occurred between 1 and 2 million years ago (Hickson 1986) and subsequently overlain by glacial till during the last Pleistocene glaciation that concluded 11,000 years ago (Clague 1981). In terms of vegetation, the study area falls within the humid lower boreal subzone (sensu Tuhkanen 1984) and in the Interior Cedar-Hemlock Biogeoclimatic Zone (ICH) of Meidinger and Pojar (1991). Hämet-Ahti (1965) gives a detailed description of the vegetation.

In their study, Gauslaa et al. (2021) recorded epiphytes on the lower living branches of 90 *Picea glauca* x *engelmannii* trees (hereafter referred to as *Picea*) in open, unmanaged mid-successional conifer forests along a 31-km north–south transect in sites representative of various forest settings including stands of the tree genera *Abies*, *Betula, Picea*, *Pinus, Populus, Pseudotsuga*, *Thuja*, and *Tsuga* in varying proportions. Studied *Picea* branches supported acidophytic chlorolichens belonging to Parmeliaceae as well as more neutrophilic cephalo- and cyanolichens from Peltigerales (Lobariaceae, Nephromataceae, Peltigeraceae, Collemataceae). The bark pH of the study branches varied from 3.9 to 6.4, while soil pH varied from 3.9 to 6.8 (Gauslaa et al. 2020). Standardized transplants of *Lobaria pulmonaria* were secured to plastic nets, which were then attached to two lower branches of each tree. The transplants were not in direct contact with tree bark but were exposed to throughfall from the overhead foliage for one year before they were harvested. Throughfall chemistry was subsequently documented by quantifying the elemental concentrations (C, N, S, Al, B, Ca, Cu, Fe, K, Mg, Mn, Mo, Na, P, and Zn) present in the *L. pulmonaria* transplants. For a comprehensive description of the study sites, methods, chemical data, along with the importance of using an indirect approach to assess throughfall chemistry, see Gauslaa et al. (2020). It is worth mentioning that conducting a direct assessment of the elemental composition in canopy throughfall poses significant challenges (Campbell et al. 2010).

The earlier studies documented the composition of the lichen-dominated epiphytic community and its associated chemical parameters using non-metric multidimensional scaling (NMDS). The NMDS1 scores demonstrated a negative correlation with bark pH and base cations (Ca, Mg, and K) and P, but a positive correlation with Mn (Supplementary Material, Table S1). These scores reflect a gradient from base cation- to Mn-rich acidic canopy throughfall, with each lichen species occupying a unique position (see Gauslaa et al. 2021).

### Lichen phylogeny of the lichens found in studied spruce canopies

Our 53 species encompass six families in three orders. At a starting point, we utilized the 7-locus megaphylogeny of Lecanoromycetes, as compiled by Nelsen et al. (2020a), with three taxonomic updates: *Pseudocyphellaria anomala,* revised to *Lobaria anomala*, *Usnea filipendula* to *U. dasopoga,* and *U. lapponica* to *U. perplexans*. We added 20 sequences to the alignment by hand, snipping out regions of the new sequences that did not match any part of the existing alignment (presumed to be ambiguously aligned regions omitted by Nelsen et al. (2020a)). Fourteen sequences of specimens seen by us were sourced from GenBank (Table 1), with priority given to specimens from western Canada. The remaining six species absent from the Nelsen et al. megaphylogeny were supplemented by unpublished ITS sequences provided by Toby Spribille at University of Alberta, Canada. These sequences were extracted from specimens included in Table 1 (methods described in Spribille et al. 2020). Following completion of the augmented alignment, we re-executed the analysis of Nelsen et al. (2020a) using their original scripts and constraints, but with our new sequences and species added. The resulting ML and bootstrap trees were subsequently pruned to include only our species using the keep.tip function in the R-package ape. We used RAxML (Stamatakis 2014) to add bootstrap values to the trimmed ML tree. Finally, we used the cophenetic function in R to compute the patristic distance (branch length) matrix for the ML tree. Our raw sequences, alignment, and modified scripts are available at 10.6084/m9.figshare.25448086.

**Table 1:** Sequences added to the megaphylogeny of Nelsen et al. (2020a)

Species	Accession	Locus	Country	Voucher
*Bryoria pikei*	KJ599553	mtSSU	Canada	Goward 05-18 (UBC)
*Bryoria pikei*	KR857122	ITS	Canada	Goward 05-20 (UBC)
*Bryoria pikei*	KR857204	MCM7	Canada	Goward 05-20 (UBC)
*Bryoria pseudofuscescens*	PP510458	ITS	Canada	Goward 21-26
*Bryoria vrangiana*	KR857150	ITS	Finland	Myllys 040811-26 (H)
*Bryoria vrangiana*	KR857232	MCM7	Finland	Myllys 040811-26 (H)
*Collema subflaccidum*	MH887481	18S	USA	?
*Hypogymnia canadensis*	MG692903	mtSSU	USA	McCune 30720
*Hypogymnia canadensis*	MG692821	ITS	USA	McCune 30720
*Hypogymnia canadensis*	MG692865	MCM7	USA	McCune 30720
*Hypogymnia dichroma*	PP510459	ITS	Canada	Goward 21-89
*Hypogymnia protea f. tessellata*	PP510460	ITS	Canada	Goward 21-60
*Hypogymnia wilfiana*	MG692946	mtSSU	Canada	McCune 31262
*Hypogymnia wilfiana*	MG692896	MCM7	Canada	McCune 31262
*Parmelia hygrophila*	PP510461	ITS	Canada	Goward 21-74
*Ramalina dilacerata*	PP510462	ITS	Canada	Goward 21-18
*Ramalina labiosorediata*	KY362420	ITS	?	Lewis 341
*Ramalina thrausta*	MN954846	ITS	Canada	Goward 99-01
*Ramalina thrausta*	MT007183	RPB1	Canada	Goward 99-01
*Usnea scabrata*	PP510463	ITS	Canada	Goward 21-40

The taxonomy of the *Sticta fuliginosa* group in Northwestern North America was revised after we had established our phylogeny (Di Meglio and Goward 2023). The sequences utilized by Nelsen et al. (2020a) for that particular species were from European material, which belongs to a different clade than the material we found in our study. The latter is now referred to as* S. fasciculata* Di Meglio & Goward. As this is the only widespread species in that group from our study area, and all members of the *fuliginosa*-group are closely related, it is unlikely that this will make a noticeable difference in our analysis.

### Characterization of phylogenetic space

One important difference between ecological and phylogenetic data is the addition of topology. Phylogenetic trees encode not only distances between nodes, but also the order in which clades branch. The techniques from cluster analysis and cladistics provide tools for inferring tree topologies from ecological data (Felsenstein 1982; Swofford and Olsen 1990) and for evaluating the correspondence of the topology of trees (Lapointe and Legendre 1992). We used patristic distance (additive distance along the branches of a phylogenetic tree between pairs of species at nodes) as a rough surrogate for age of evolutionary divergence (Brown and Smith 2017). Because we are not concerned with the topology of the tree, but only with the depth of the divergence, direct comparison of our phylogenetic data (the patristic distance matrix) and ecological data via standard ordination analyses and correlation tests from the ecological literature should be valid for present purposes.

The structure of the distribution of species in ordination space is notably different from their distribution in ecological space. Instead of forming clouds of closely related species, we found an intriguingly geometric arrangement of nested radiating tetrahedra (Supplementary Material Fig. S1). At any given taxonomic level, the groups at the next lower level radiate out orthogonally, with their subgroups in turn radiating orthogonally, and so on. Presumably this pattern is an artefact of the structure of the bifurcating phylogenetic tree, which precludes any horizontal mixing between species on clades that diverged earlier in history. In actual practice, species of any taxonomic group can be expected to evolve new adaptations toward occupying any ecological niche, resulting in a diffusion in all directions in ecological space, not just movement farther down a branch. Any departure from a normal-distributed, symmetric distribution in ecological space reveals the presence of an interaction with external forces. The abnormal structure of the phylogenetic PCoA space reveals the artificial constraints enforced by the structure of the bifurcating phylogenetic tree.

### Statistical analyses

To investigate the ecological relationships among the species, we performed a global non-metric multidimensional scaling (gNMDS) with two dimensions based on a Jaccard distance matrix derived from the presence–absence data of 53 lichen species on the lower branches of 90 examined *Picea* canopies. This was done using the default settings of the metaMDS wrapper-function of the vegan R-package (Oksanen et al. 2022). The Jaccard distance, a frequently employed metric for ecological community analyses, was selected owing to its suitability for binary data and for discriminating degree of shared species composition across sites. The stress value was 0.164. We assessed the significance of measured environmental variables, including elemental concentrations of C, N, S, Al, B, Ca, Cu, Fe, K, Mg, Mn, Mo, Na, P and Zn; bark-, and soil-pH, tree height, circumference at breast height, basal area, and percent open sky on the ordination using the envfit function with 999 permutations. The ordination of species and the environmental variables as vectors is given in Fig. S2.

To enable quantitative statistical comparisons of the phylogeny and ecology of the species, we performed a principal coordinates analysis (PCoA) of the patristic distance (additive branch length) matrix using the cmdscale function of the R-package stats. This analysis situates each species in a Euclidean space in which the distances between species closely approximate their degree of relatedness in the phylogenetic tree, analogous to the ecological space produced by the gNMDS analysis of Jaccard distances above.

We used procrustean randomization tests (PROTEST; Peres-Neto and Jackson 2001) to evaluate the concordance between the PCoA of the patristic distances and the gNMDS of the ecological distances, using 9999 permutations. To investigate the relationship between the distribution of lichen species in phylogenetic space and across environmental gradients, we derived gNMDS scores corresponding to these gradients. This involved adjusting the orientation of the ecological ordination using the MDSrotate function, aligning the first axis with each environmental variable showing significant correlation as identified by the envfit function (Table S1, Fig. S2). Subsequently, we calculated the species scores along this axis for each rotated configuration. These scores, normalized to a mean of 0 and a standard deviation of 1, measure the position of each species along the environmental gradients. We used these normalized scores in Pearson correlations against the first dimension of the phylogenetic PCoA, which corresponds to the axis between Peltigerales and Lecanoraceae, our best represented groups, to understand how phylogeny influences species distribution along environmental gradients. To validate the robustness of the first PCoA dimension against variation in species inclusion, and thereby confirm its representation of a true gradient in relatedness, we randomly omitted ten species from the matrix and repeated this process 100 times, each time recalculating the PCoA. The first PCoA dimension of the complete dataset was then correlated with the first dimension from each of the 100 iterations. The high average *R*^2^-value of 0.998 (lowest is 0.962) across these correlations supports the stability and reliability of the original PCoA dimension, regardless of species inclusion.

## Results

**Fig. 1:**
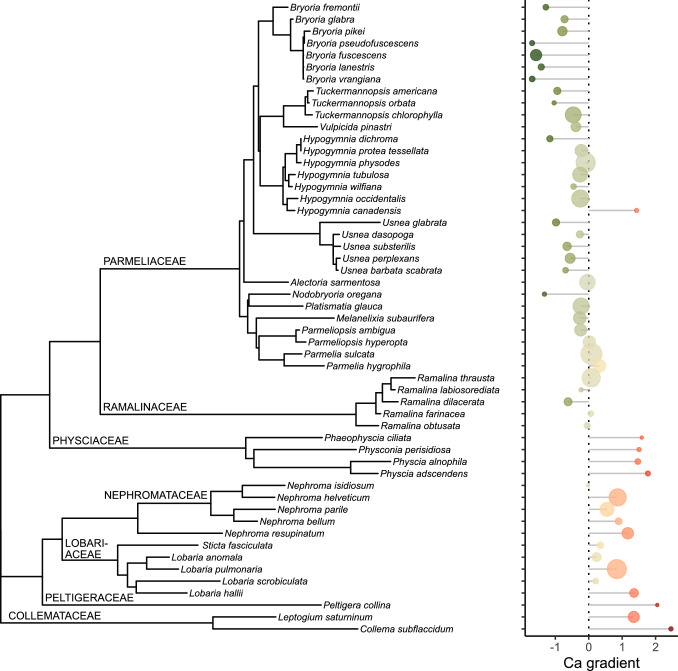
A phylogenetic tree of all macrolichen species found on sampled lower branches of 90 *Picea glauca x engelmannii* canopies in south-central British Columbia. The families ranked from top to bottom are as follows, Parmeliaceae (31 spp.), Ramalinaceae (5 spp.), Physciaceae (4 spp.), Nephromataceae (5 spp.), Lobariaceae (5 spp.), Peltigeraceae (1 sp.), and Collemataceae (2 spp.). The accompanying lollipop plot to the right visualizes the Ca gradient where 0 represents the average value. The intensity of the colors corresponds to the length of the horizontal bars, thereby reflecting individual Ca-scores. The size of the circles represents the frequency of each species in sampled tree canopies

The phylogenetic tree, encompassing all macrolichens found on lower branches of *Picea* canopies in south-central British Columbia (Fig. 1) comprised the Lecanorales families Parmeliaceae (31 spp.) and Ramalinaceae (5 spp.), the Caliciales family Physciaceae (4 spp.), and the Peltigerales families Nephromataceae (5 spp.), Lobariaceae (5 spp.), Peltigeraceae (1 sp.), and Collemataceae (2 spp.). These taxa formed radiating clusters in a 3-D PCoA of the patristic distance matrix (Supplementary Material, Fig. S1), each cluster representing a single family. The families of Peltigerales grouped closely together, forming one point of a tetrahedron, the other three points being formed by the Parmeliaceae, Physciaceae and Ramalinaceae, respectively. Each of these four groupings was roughly equidistant from the others. The first axis, which accounts for 42.7% of the variation, and has been validated as a consistent and reliable measure with a high average *R*^2^-value of 0.998 from our robustness tests, aligns mostly with the axis connecting the four Peltigeralean families and Parmeliaceae, our most well represented groups. This underscores the argument for its use as a primary axis in representing the relatedness among these macrolichen species. While the Ramalinaceae and Physciaceae align more with the second and third axes respectively, their positions along the first axis are still important in manifesting their degrees of relatedness.

The procrustean association metrics showed a significant concordance between the two-dimensional spaces of phylogeny and ecology (*m*_12_^2^ = 0.5887; *r* = 0.6413; *P* < 0.001). The patristic distance matrix between all species showed correlations with the respective matrices of ecological distances (Table 2). Calcium was identified as the environmental factor most strongly correlated with phylogeny, although factors positively (bark and soil pH, K, Mg) or negatively (Mn) associated with Ca in forest ecosystems were also highly significant (Table 2). Hence, the lollipop plot displaying the Ca-scores for each species was aligned with the phylogenetic tree (Fig. 1). The species of Parmeliaceae were mostly associated with negative Ca-scores, indicating lower than average Ca concentrations in the throughfall from the surveyed canopies. Notably, the rare *Hypogymnia canadensis* (*n* = 2) emerged as the only outlier within this family. The most common Parmeliaceae species, namely *Parmelia sulcata* (*n* = 74)*, Hypogymnia physodes* (*n* = 61)*,* and *Alectoria sarmentosa* (*n* = 39), exhibited Ca-scores near 0, which is the overall average. Among the frequent Parmeliaceae species, only *Parmelia hygrophila* (*n* = 25) had a marginally positive score. The genera *Bryoria, Nodobryoria, Tuckermannopsis,* and *Usnea* had the lowest Ca-scores. The members of the Ramalinaceae were associated with intermediate Ca-scores.

**Table 2:** Pearson correlation coefficient (*r*) between the first axes of the phylogenetic and ecological ordination of species and environmental parameters, respectively

Parameter	*r*	*P*
Ca	0.718	< 0.001
Mn	−0.694	< 0.001
NMDS1	−0.681	< 0.001
Percent open sky	−0.679	< 0.001
Bark pH	0.668	< 0.001
Basal.area	0.663	< 0.001
C	−0.661	< 0.001
Soil pH	0.648	< 0.001
K	0.647	< 0.001
Mg	0.638	< 0.001
NMDS2	0.526	< 0.001
Tree height	−0.437	0.001
P	0.364	0.007
Trunk circumference	−0.338	0.013

Elements reflect the canopy throughfall chemistry monitored as the elemental concentration in standardized transplants of *Lobaria pulmonaria* on lower branches of *Picea glauca* x *engelmannii* exposed to canopy throughfall for one year. NMDS1 and NMDS2 refer to the first and second axis of the original gNMDS. Before calculation of an *r*-value, the ordination was rotated for each factor to maximum variation along the first axis

Whereas Lecanorales members (Parmeliaceae, Ramalinaceae) had Ca-scores ≤ 0, Caliciales (Physciaceae) and Peltigerales species consistently exhibited positive Ca-scores (Fig. 1). The highest Ca-scores were observed in Physciaceae, Collemataceae, and Peltigeraceae. In contrast, Nephromataceae and Lobariaceae had lower, yet consistently positive Ca-scores. Consequently, the strongest contrast in Ca-scores was seen between Lecanorales on one side and the group including Caliciales (Physciaceae) and Peltigerales on the other.

**Fig. 2:**
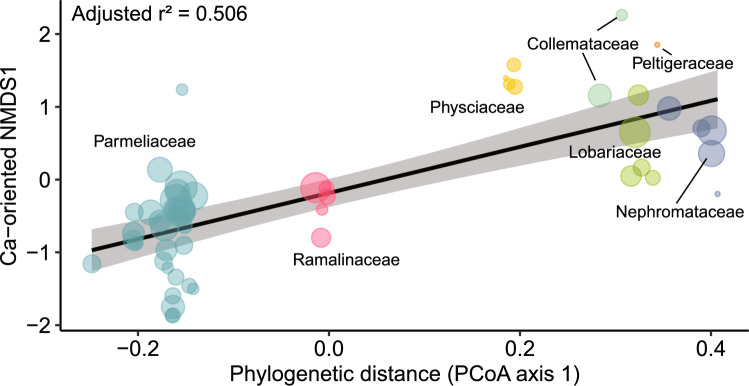
Relationship between Ca-oriented NMDS1 and phylogenetic distance across lichens found in 90 *Picea glauca* x *engelmannii* canopies in south-central British Columbia. This relationship is depicted as a regression line with standard errors represented as a shaded band. The equation for this regression line is Ca-oriented NMDS1 = −0.1837 + 3.1681 × (phylogenetic distance); *r*^2^_adj_ = 0.506; *P* = 1.47 × 10^–9^; *df* = 51. The x-axis represents the primary variation in patristic distances among different lichen species, as derived from the Principal Coordinates Analysis. The distance between species along this axis reflects the degree of phylogenetic relatedness. The distance between families corresponds roughly to the age or depth of the phylogenetic split between the families. The size of the circles represents the frequency of the species in sampled tree canopies

The Ca-scores of species (as shown in Fig. S 2B) correlated highly significantly with the first component of the PCoA of the patristic distance matrix based on phylogenetic differences (Fig. 2). This first component, which is a measure of phylogenetic distance, accounted for 42.7% of the variation of the PCoA. The significant relationship in Fig. 2 was primarily due to family-specific Ca-oriented NMDS1-scores, with no discernible relationship observed within families. Notably, the Physciaceae family of the Caliciales order, had some of the highest Ca-scores, despite its phylogenetic position between Lecanorales and Peltigerales.

## Discussion

### The link between lichen phylogeny and chemistry

The epiphytic vegetation composed of cyano- and cephalolichens from Peltigerales typically colonizes trees with bark characterized by relatively high base cation content and pH. On the other hand, species-rich chlorolichen assemblages in Lecanorales (family Parmeliaceae) thrive on trees with acidic bark. This broad phylogenetic-chemical correspondence has been observed in Norwegian *Quercus*- (Gauslaa 1985) and *Picea*-dominated forests (Gauslaa and Holien 1998), as well as in Canadian *Picea* forests (Goward and Arsenault 2000). Our primary contribution consists in quantifying the strong link between Ca-availability in tree canopies and large-scale phylogeny across orders or families of epiphytic macrolichens. The depth of this correspondence probably implies an ancient adaptation of lichens to different chemical environments during the Cretaceous period (145–66 million years bp) when many species of angiosperm trees appeared (Crane et al. 1995) and Parmeliaceae and Peltigerales diversified (Amo de Paz et al. 2011; Widhelm et al. 2021).

At finer taxonomic scales, our findings further suggest that the smaller species-specific variations in Ca-scores within Lecanorales and Peltigerales reflect more recent chemical specializations. For instance, within the mostly oligotrophic Parmeliaceae (Lecanorales), the recently evolved genus *Bryoria* (Divakar et al. 2017) dominates tree branches with the most acidic bark (bark pH < 4.0; Marmor and Randlane 2007), consistent with their very low Ca-scores. Interestingly, the photobiont found in the majority of *Bryoria* species is *Trebouxia simplex* (Lindgren et al. 2014). This particular photobiont is significantly associated with the most acidic rocks in saxicolous lichen communities (Peksa et al. 2022). Similarly, *Lobaria scrobiculata*, characterized by one of the lowest Ca-scores in Peltigerales, is known to thrive on more acidic bark (pH ≥ 4.2) than other *Lobaria* species (pH ≥ 4.6; Gauslaa 1985).

### The chemical gradient

The Ca-scores of the studied lichens reflect local canopy throughfall chemistry, and are positively correlated with Mg, K, soil and bark pH, and negatively with Mn. These factors contribute to the first component in non-metric multidimensional scaling ordinations encompassing either the epiphytic vegetation found in *Picea* canopies or the vascular vegetation on the ground beneath these canopies (Gauslaa et al. 2021). At the same time, concentrations of Mn (Richardson 2017) and base cations (Reich et al. 2005) in tree tissues, which contribute to canopy throughfall chemistry in the form of leachates, exhibit a correlation with the respective concentrations in the underlying soil. Thus it can be seen that Ca and strongly correlated elements of epiphytic lichens are linked to a complex forest soil gradient from acidic and oligotrophic to less acidic mesotrophic environments (Giesler et al. 1998).

The strong interrelationships between chemical factors make it challenging to pinpoint a single causal link explaining the robust correlation between Ca-scores and phylogeny. Ca is an essential micronutrient for fungi and algae (Pitt and Ugalde 1984). Mn, another essential micronutrient (Hauck and Paul 2005), exhibits a strong yet negative relationship with Ca (Supplementary Material Fig. S2). While Mn plays a vital role in the water-splitting reaction in photosystem II (PSII) (Dau and Haumann 2008), it often occurs in high concentration in natural, unpolluted forest soils, posing toxicity threats to plants (St Clair and Lynch 2005) and lichens (Hauck and Paul 2005). Excess Mn can impair the viability of symbiotic lichen diaspores (Hauck et al. 2002b) and diminish the effective quantum yield of PSII (Hauck et al. 2003; Hauck and Paul 2005), especially in Peltigerales (Hauck et al. 2006). By contrast, the acidophyte *Hypogymnia physodes* (Parmeliaceae), a species that thrives on Mn^2+^-rich bark (Hauck and Spribille 2005), employs carbon-based secondary compounds (CBSC) to minimize internal uptake of Mn^2+^ (Hauck 2008). The detrimental effects of Mn on lichens are mitigated by high levels of Ca and Mg (Hauck et al. 2002a; 2006). The high Ca levels are crucial for the presence of Peltigerales species on *Quercus* bark (Gauslaa 1985; Farmer et al. 1991), though not necessarily on other deciduous tree species (Gauslaa 1995). Furthermore, the growth of *Lobaria pulmonaria* (Peltigerales) correlates positively with bark pH (Gauslaa and Goward 2012) and with Ca content in throughfall from *Picea* canopies (Gauslaa and Goward 2020). Similarly, the optimal quantum yield of PSII in *L. pulmonaria* increases with pH (Gauslaa et al. 1996). At the same time, the observation that the most Ca-rich throughfall from *Populus* canopies does not necessarily augment lichen growth suggests a possible saturation effect of high Ca-concentrations (Gauslaa and Goward 2020).

The members of Peltigerales contain significantly more K^+^ than Lecanorales species (Beckett et al. 2003), suggesting that they may benefit from the higher K concentration typical of Ca-rich canopy throughfall (Gauslaa et al. 2020). The availability of P, which is more weakly correlated with Ca (Supplementary Material Fig. S2), increases as soil pH rises in more or less acidic soils (Penn and Camberato 2019). The lack of P sometimes limits growth of epiphytic Peltigerales species (Benner et al. 2007; McCune and Caldwell 2009; Benner 2011), but not always (Gauslaa and Goward 2012; Marks et al. 2015). The complex oligotrophic-mesotrophic gradient shape the relative dominance of Lecanoralean versus Peltigeralean or Calicialean lichens, though the specific causal element might differ across habitats.

### Functional traits separating Peltigerales and Lecanorales

Significant functional disparities exist between members of Peltigerales and Lecanorales (Kraichak et al. 2018), which often influence elemental uptake or heavy metal detoxification. For species of Peltigerales, the primary (cyanolichens) or the secondary photobionts (cephalolichens) are N-fixing cyanobacteria, while epiphytic genera of Lecanorales (chlorolichens) only host green algal photobionts (Nelsen et al. 2020a).

The chitin in Peltigeralean cell walls is more abundant than in Lecanoralean lichens (Boissière 1987; Honegger and Bartnicki-Garcia 1991; Sundberg et al. 1999; Palmqvist et al. 2002)—an observation that correlates with the documented ability of chitin to bind cations (Galun et al. 1983). At the same time, chitin contributes to the binding of heavy metals in cell walls (Galli et al. 1994) and potentially enhances the fungal function as a biosorbent (Burford et al. 2003).

CBSCs in lichens serve various functional roles (Solhaug and Gauslaa 2012), including heavy metal tolerance and uptake (Purvis et al. 1987; Purvis and Pawlik-Skowronska 2008; Hauck et al. 2009; 2013). The Lecanorales family Parmeliaceae is high in CBSCs (Ahti et al. 2011), while most Peltigeralean lichens, especially cyanolichens, either lack these substances or produce them in low concentration (Ahti et al. 2007). Epiphytic lichens containing depsides, depsidones, or usnic acid, are usually found on acidic bark, while species that lack them thrive on bark with higher pH (Paukov et al. 2022).

Finally, Peltigeralean and Lecanoralean species also differ in other traits, with the former containing higher levels of water-soluble phenolic compounds (Zagoskina et al. 2013), higher water contents at full turgor, elevated rates of extracellular superoxide production (Beckett et al. 2003), higher laccase activity (Laufer et al. 2006), and higher concentration of chlorophyll (Palmqvist et al. 2002) and K^+^ (Beckett et al. 2003). It is not clear whether these traits play roles in chemical adaptations.

### Epiphytic Peltigerales and Lecanorales species tend to be mutually exclusive

**Fig. 3:**
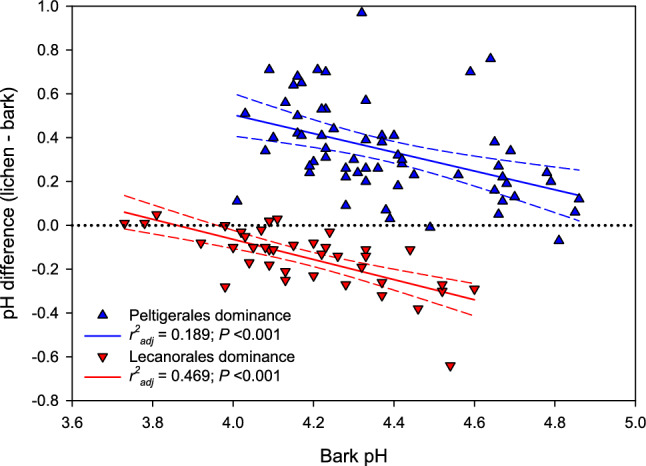
Relationship between the pH difference between the lichen vegetation itself and the underlying bark versus the bark pH on *Picea abies* branches in boreal rainforests in central Norway. Figure 3 is based on data from *Picea abies* branches in Norwegian boreal rainforests (Gauslaa and Holien 1998) that share many species in common with studied forests in British Columbia. Hatched lines show the 95% confidence interval, the dotted line is the 0-line

*Picea abies*-dominated boreal rainforests in Norway (DellaSala et al. 2011b) share floristic and ecological characteristics with forests in British Columbia (DellaSala et al. 2011a). In both regions, the species of Peltigerales or Lecanorales tend to be mutually exclusive on *Picea* branches, a phenomenon seemingly connected to measured pH contrasts of the bark and lichens (Fig. 3; data sourced from Gauslaa and Holien 1998). Upon plotting the pH contrasts between the lichens and the underlying tree bark against the bark pH, it is observed that members of Lecanorales consistently exhibit lower pH than the adjacent tree bark. Conversely, Peltigerales species are less acidic than the tree bark (Fig. 3). The regression line for Lecanorales lichens intersects the 0-line at a pH of 3.9, which seems to represent a pH-equilibrium between these epiphytes and the tree bark. On the other hand, a higher pH level of around 5.2 signifies the corresponding equilibrium for Peltigerales. This distinct segregation of lichen–bark pH-relationships for Lecanorales and Peltigerales (Fig. 3) aligns with the clear separation of their respective Ca-scores (Fig. 1). Such findings support the conclusion that Peltigerales and Parmeliaceae in Lecanorales exhibit fundamental chemical differences, even when found on different continents.

### Lichens as chemical players

The indirect method of estimating the chemistry of canopy throughfall, using *L. pulmonaria* transplants, can be verified by comparing the retention efficiencies of Peltigerales and Lecanorales members in capturing various elements from canopy throughfall. Asplund et al. (2015) conducted a comparable study of elemental composition in transplants of the acidophytic *Hypogymnia physodes*, placed on trunks of conifers and broadleaved deciduous trees. Their observations revealed significantly higher base cation concentrations and lower Mn concentrations beneath deciduous trees. These findings align with the results of our study, thereby reinforcing the conclusion that lichen transplants can provide a reliable measure of canopy throughfall chemistry.

The contrasting Ca-scores observed in Lecanorales and Peltigerales (Fig. 1) along with significantly different group-specific relationships between the pH of lichens themselves and the pH of their tree bark (Fig. 3), suggest that epiphytic Lecanoralean lichens may reduce the bark pH, while Peltigeralean species may increase it. The ability of a lichen fungus to acidify its micro-environment, as demonstrated by Burgstaller and Schinner (1993), aligns with data for Parmeliaceae, but does not apply to members of the Peltigerales, which have a higher pH than their substrate (Gauslaa and Holien 1998). Moreover, in lichen-dominated soil crust communities, certain species have been found to increase soil pH while others reduce it (Concostrina-Zubiri et al. 2013; Ghiloufi et al. 2023). This suggests that lichens may not passively adjust to their chemical environment but could play more active chemical roles.

## Conclusions

Well-documented differences in the ecological niches occupied by epiphytic lichens with respect to substrate chemistry are shown to be deeply rooted in lichen fungal phylogeny. Such deep-rooted adaptations to secure essential mineral nutrients in extreme chemical environments in tree canopies probably account for earlier reported transferability across continents of lichens as bioindicators (Delves et al. 2023). Applied more broadly, the findings reported here suggest that recognition of lichen genera or families alone can enable reliable assessment of habitat chemistry.

## Supplementary Information

Below is the link to the electronic supplementary material.

Supplementary file1 (DOCX 747 KB)

## Data Availability

Raw sequences, alignment, and modified scripts are available at 10.6084/m9.figshare.25448086.
