# Supplementary material for: Unraveling the interplay between phylogeny and chemical niches in epiphytic macrolichens
Source: Oecologia. 2024 Dec 7;207(1):4. doi: 10.1007/s00442-024-05641-9 (PMC11625070; doi:10.1007/s00442-024-05641-9)
Supplement: Supplementary file 1 — Supplementary file1 (DOCX 747 KB) [file 442_2024_5641_MOESM1_ESM.docx]

**Supplementary Material**

**Table S1**. Results of fitting environmental variables to the gNMDS ordination shown in Fig. S2, using the envfit function in vegan. The table shows the squared correlation coefficients (*r*^2^) and the *P*-values for each variable. The variables are ordered by decreasing *r^2^* values, and thus decreasing correlation between the variable and the ordination space. The *P*-values are based on 999 permutations.

| Variable | *r^2^* | *P* |
| --- | --- | --- |
| Bark pH | 0.528 | <0.001 |
| Mg | 0.477 | <0.001 |
| Percent open sky | 0.431 | <0.001 |
| K | 0.390 | <0.001 |
| Trunk circumference | 0.382 | <0.001 |
| Ca | 0.274 | <0.001 |
| Mn | 0.249 | <0.001 |
| Tree height | 0.245 | <0.001 |
| Basal area | 0.213 | <0.001 |
| Soil pH | 0.191 | <0.001 |
| C | 0.173 | <0.001 |
| P | 0.097 | 0.008 |
| Al | 0.038 | 0.179 |
| Fe | 0.036 | 0.208 |
| Cu | 0.035 | 0.204 |
| Mo | 0.034 | 0.226 |
| Zn | 0.018 | 0.482 |
| B | 0.005 | 0.819 |
| Na | 0.001 | 0.952 |
| S | 0.001 | 0.959 |


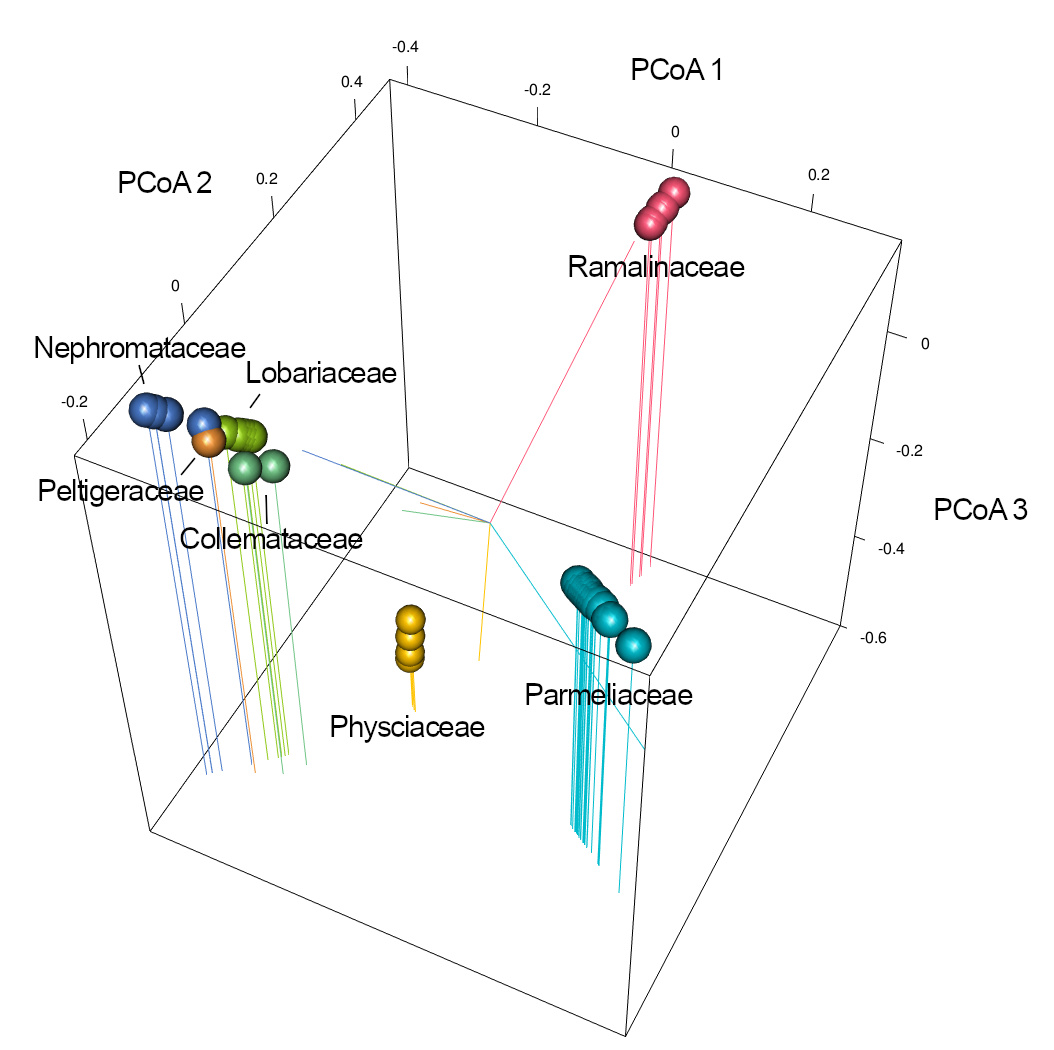


**Figure S1.** First three dimensions of principle coordinates analysis ordination (PCoA) of the patristic distance matrix built from the 7-locus phylogenetic tree adapted from Nelsen et al. (2020). Colored dots represent individual species, their color indicating the family.

**A**


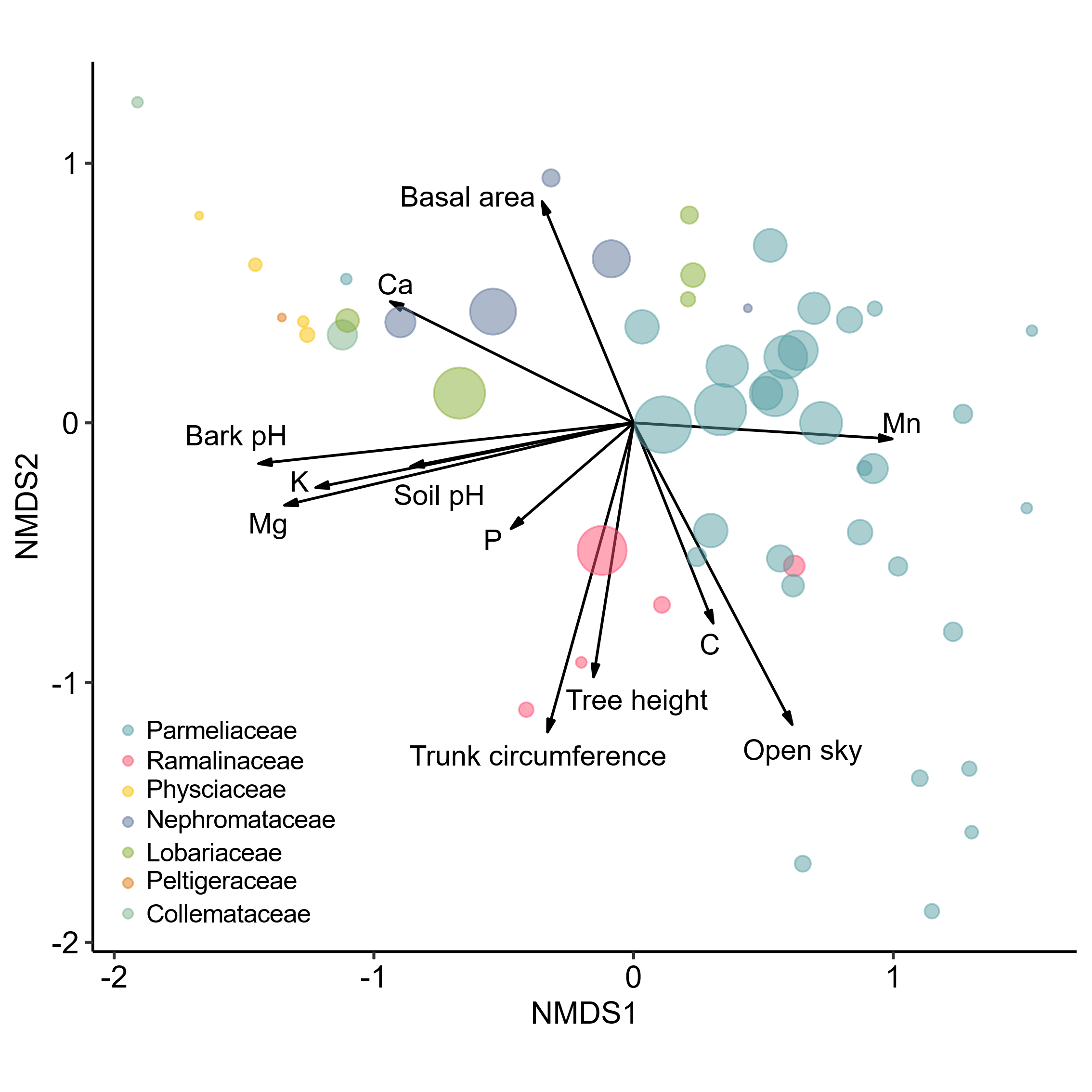


**B**


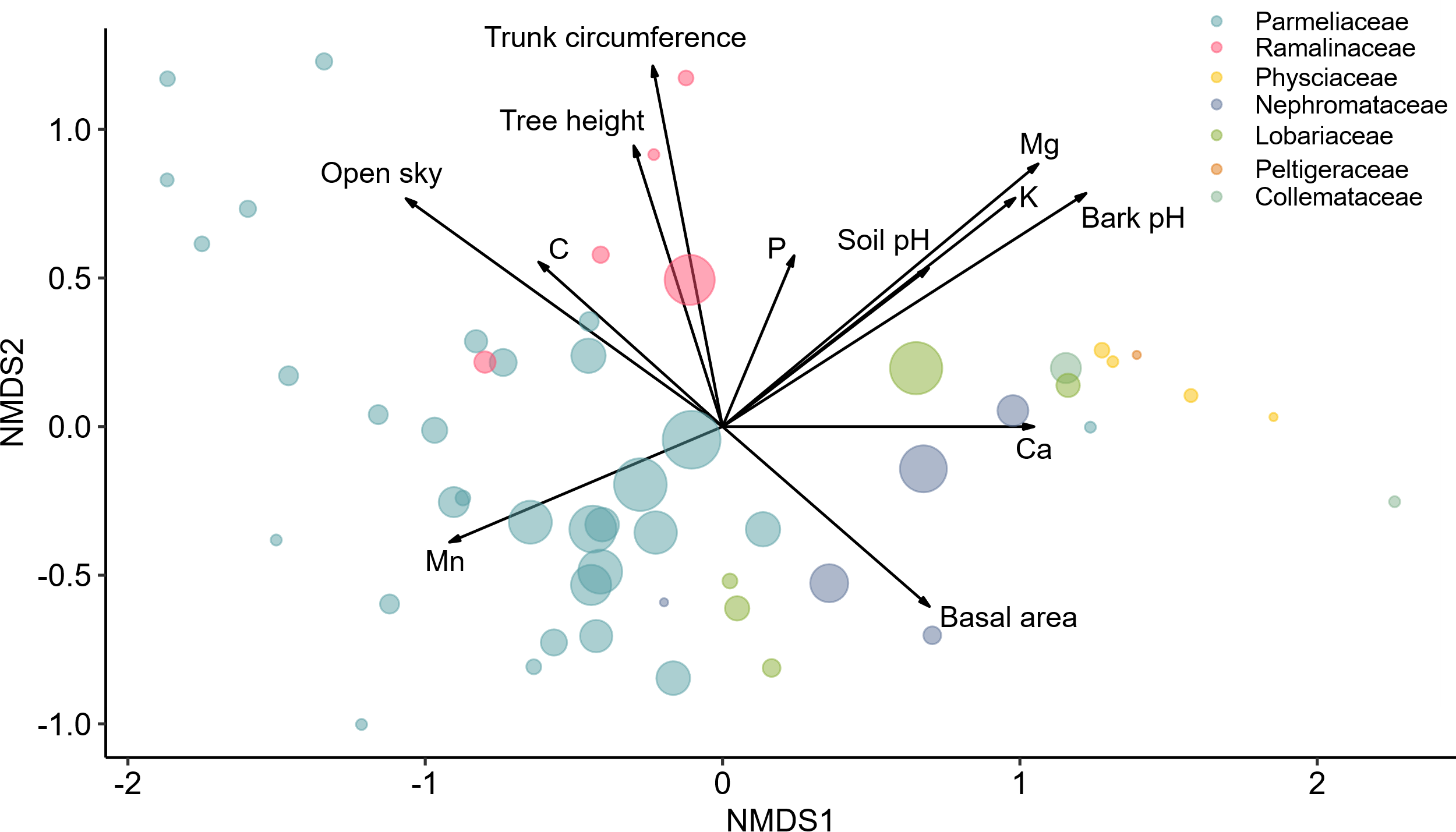


**Figure S2. A**: Global non-metric multidimensional scaling (gNMDS) ordination plot of the lichen community composition on the branches of *Picea glauca x engelmannii*. Colored dots represent individual species, their color denote lichen family, and their size are relative to their frequency in the dataset. The arrows show the direction of increasing environmental variables and their length are proportional to the correlation between the variable and the ordination. Only significant (*P* < 0.05) vectors are included. This is a modified ordination after (Gauslaa et al., 2021) after excluding the bryophyte species and adding the information of species frequency. **B:** The same ordination as in **A** but rotated along the Calcium (Ca) axis.

**References:**

Gauslaa Y, Goward T, Asplund J (2021) Canopy throughfall links canopy epiphytes to terrestrial vegetation in pristine conifer forests. Fungal Ecology 52:101075. doi:<https://doi.org/10.1016/j.funeco.2021.101075>

Nelsen MP, Lücking R, Boyce CK, Lumbsch HT, Ree RH (2020) The macroevolutionary dynamics of symbiotic and phenotypic diversification in lichens. PNAS 117 (35):21495-21503. doi:10.1073/pnas.2001913117
